# Sex differences in the association between self-reported sleep duration, insomnia symptoms and cardiometabolic risk factors: cross-sectional findings from Brazilian longitudinal study of adult health

**DOI:** 10.1186/s13690-020-00429-8

**Published:** 2020-05-29

**Authors:** Aline Silva-Costa, Lucia Rotenberg, Aline A. Nobre, Dora Chor, Estela M. Aquino, Enirtes C. Melo, Sandhi M. Barreto, Maria Inês Schmidt, Rosane H. Griep

**Affiliations:** 1 Department of Collective Health, Federal University of Triangulo Mineiro (UFTM), Uberaba, Minas Gerais, Brazil; 2 Laboratory of Health and Environment Education, Oswaldo Cruz Institute – Fiocruz, Rio de Janeiro, Brazil; 3 Scientific Computing Program, Oswaldo Cruz Foundation – Fiocruz, Rio de Janeiro, Brazil; 4 National School of Public Health, Oswaldo Cruz Foundation - ENSP/FIOCRUZ, Brazil, Rio de Janeiro, RJ Brazil; 5 Institute of Collective Health, Federal University of Bahia, Salvador, Brazil; 6 Postgraduate Program in Public Health and School of Medicine & Clinical Hospital, Universidade Federal de Minas Gerais, Belo Horizonte, MG Brazil; 7 Postgraduate Programme in Epidemiology - School of Medicine, Federal University of Rio Grande do Sul, Porto Alegre, RS Brazil

**Keywords:** Sleep duration, Insomnia, Hypertension, Obesity and glycated hemoglobin

## Abstract

**Background:**

The U-shaped associations between sleep durations and cardiometabolic risk factors (glycated hemoglobin levels, obesity, *hypertriglyceridemia*, hypertension and cholesterol levels) are still inconclusive. Moreover, as sleep is comprised of quantitative and qualitative aspects, exploring both insomnia symptoms and sleep duration are relevant when evaluating the potential effects of sleep problems on health. The aim was to evaluate sex-specific associations between sleep problems and cardiometabolic risk factors.

**Methods:**

This cross-sectional study used data from wave two of the Brazilian Longitudinal Study of Adult Health (ELSA-Brasil), including 7491 women and 6232 men. Questionnaires were administered to provide information about socioeconomic conditions, lifestyle, and sleep characteristics. A 12-h fasting blood sample was drawn to measure serum cholesterol, triglycerides, and glycated hemoglobin. Blood pressure, weight and height were also measured using standard equipment. Generalized additive models were used to evaluate the curve shape of the relationship between self-reported sleep duration and the outcomes. Logistic regression was performed to investigate the magnitude of the associations of self-reported sleep duration, insomnia symptoms, and short sleep plus insomnia symptoms with cardiometabolic risk factors.

**Results:**

For women, self-reported sleep duration and insomnia symptoms (either separately or linked to short sleep duration) were associated with obesity, hypertension and glycated hemoglobin after adjusting for the confounders. The magnitudes of the associations between self-reported short sleep duration plus insomnia symptoms and the outcomes were slightly increased, considering sleep duration or insomnia symptoms separately. For men, both long sleep duration and insomnia symptoms were associated with hypertriglyceridemia after adjusted for the confounders.

**Conclusion:**

These findings suggest possible sex-specific patterns, since obesity, hypertension and high glycated hemoglobin were associated with self-reported sleep duration and insomnia symptoms in women, but not in men, and reinforce the importance of considering quantitative and qualitative aspects of sleep for the prevention and management of the outcomes.

## Background

Studies into sleep have increased in recent decades, since it was recognized as a public health issue due to the possible negative effects of sleep problems on health. Previous studies have suggested a link between sleep duration and diabetes, obesity, hypertension and metabolic syndrome [1–9]. Findings related to sleep duration and plasma lipids are scarce and less consistent [10–12], Although some studies have found that both short and long sleep durations are associated with diabetes [5, 7, 11, 12], hypertension and obesity [11, 12], other studies did not observe this U-shaped relationship [3, 12–16]. Moreover, the sleep duration range that corresponds to the lowest risk of cardiometabolic diseases remains unclear. In fact, the wide variation in the reference category (*i.e,* adequate sleep) across studies (from > 6 h to < 9 h) [3, 5, 6, 17] clearly shows that the definition of the reference category deserves attention.

Concerning insomnia symptoms (difficulty initiating sleep, difficulty maintaining sleep, early morning awakening), the studies related to cardiometabolic issues are less extensive [3], compared to the ones related to sleep duration. Having difficulty falling or staying asleep (‘all of the time’ or ‘most of the time’) was associated with type 2 diabetes in young female registered nurses [18], and with both hypertension and dyslipidemia among public sector employees [19]. Meta-analysis studies showed that the risk of type 2 diabetes [7] and hypertension [3] were, respectively, 84 and 20% higher for subjects who reported difficulties in maintaining their sleep [3, 7]. In a meta-analysis including studies on insomnia and obesity published in the past 10 years, the authors stated that associations are weak, particularly for European samples [19]. These findings stress that there is no single pattern of results and reinforce the need for studies with other population groups.

As sleep is comprised of quantitative and qualitative aspects, exploring both insomnia symptoms and sleep duration are relevant when evaluating the potential effects of sleep problems on health. In this context, the interaction between short sleep duration and insomnia symptoms, which leads to increased health risks has also been discussed [20, 21]. Cai et al. [22] found a significant association between insomnia symptoms and obesity among participants with short sleep duration, but not among those who reported normal sleep duration (6–8 h). Similarly, short sleep duration with insomnia symptoms was associated with hypertension [23]. In relation to diabetes, an association was observed in those with chronic insomnia plus short sleep duration, but not among those with insomnia symptoms plus short sleep duration [24].

The above-mentioned associations do not specifically comment on the possible different effects between men and women, despite the known gender and sex differences in relation to sleep problems [25–27]. Few studies have investigated sex-specific associations between sleep and negative outcomes related to non-communicable chronic diseases [5]. Gender differences have been detected in studies of sleep problems, with significant associations in women (but not men) with regard to hypertension [28], the prevalence of dyslipidemia [29], and obesity [30]. Short sleep duration was associated with a greater BMI and waist circumference among women but not among men [31]. However, as pointed out by Theorell-Haglöw et al. [32] research on gender differences in sleep medicine appears to be limited. In a meta-analysis study on sleep and type 2 diabetes, Shan et al. [5] emphasize the relevance of data on sex, which were usually limited in the original studies. These data reinforce the need for testing the hypothesis of sex-differences in the association between sleep patterns and cardiometabolic factors.

The mechanisms involved in the relationships between sleep problems and metabolic disorders include reduced glucose tolerance and compromised insulin sensitivity [1]. Insulin and glucose alterations associated with sleep restriction may be partially mediated by neuroendocrine changes, including high evening cortisol levels and increased sympathetic nervous system activity and catecholamine production [33]. Nocturnal sleep deprivation can lead to abnormal cortisol release during the night, which may result in decreased sensitivity to insulin in the morning. Elevations in cortisol and increased sympathetic nervous system activity are also related to impaired vasodilatation and hypertension. Short sleep duration also reduces heart rate variability [33], which highlights the importance of sleep in the regulation of many cardiometabolic functions [1].

Given all the gaps related to the associations of sleep duration and insomnia symptoms with cardiometabolic diseases, and considering that hypertension, obesity, hyperglycemia and dyslipidemia can lead to cardiovascular disease, the leading non-communicable diseases in terms of premature deaths [34], this investigation may provide relevant evidence concerning public health priorities. Therefore the objective of this study was to investigate whether there are sex-specific patterns in the association of cardiometabolic risk factors with (i) self-reported short sleep duration, (ii) self-reported long sleep duration, (iii) insomnia symptoms, and (iv) the combination of insomnia symptoms and self-reported short sleep duration.

## Methods

### Study population and setting of the study

This cross-sectional study used data from wave two (2012–2014) of the Brazilian Longitudinal Study of Adult Health (ELSA-Brasil) [35], which was a multicenter study following civil servants aged 35 to 74 yrs. old who were recruited between August 2008 and December 2010 (first wave) at public institutions in six Brazilian state capitals (Salvador, Belo Horizonte, Rio de Janeiro, São Paulo, Vitória and Porto Alegre). The second wave re-examined and interviewed 14,014 individuals. Wave 2 included data on sleep patterns (not evaluated in wave 1), which allowed us to perform the current analyses, including 13,722 participants (7491 women and 6231 men) with complete data for all variables. A total of 292 participants with missing data on variables related to self-reported sleep duration (*n* = 98), insomnia symptoms (*n* = 97) and outcomes (*n* = 42) was excluded. Participants who reported sleep durations < 3 h or > 12 h were also not considered in the analyses (*n* = 29 women and 26 men).

### Variables definition

A comprehensive set of questionnaires was administered to provide detailed information on socioeconomic conditions, habits, lifestyle, and health. The study design, sampling procedures, construction of the questionnaire and quality assurance and control measures were previously detailed [36, 37].

#### Sleep variables

##### Self-reported sleep duration

The self-report questionnaire included the question “How many hours of sleep do you get in a usual night’s sleep? |__|__| hours |__|__|minutes” [14, 21, 28] Participants were classified into three groups according to the exact sleep duration they reported: the short sleep duration group (≤ 6 h), adequate sleep duration group (> 6 h and ≤ 8 h) and long sleep duration (> 8 h).

##### Insomnia symptoms

The following questions were asked: “In relation to your sleep during the night, at home, during the last 4 weeks, how often did you have difficulty in falling asleep?”, “... wake up and have difficulty going to sleep again?”, and “... wake up before the desired time and not manage to sleep again?“ [21, 25] Participants who answered never, rarely or sometimes were classified as no complaint. Participants who answered almost always or always were classified as having a complaint. Participants who reported any of the three described complaints were assigned to the insomnia group.

Self-reported short sleep duration plus insomnia symptoms: this variable considered the combination of self-reported sleep duration and insomnia symptoms. We could not create a “long sleep duration + insomnia” category because few participants (18 men and 45 women) were classified in this group. Therefore, participants with short self-reported sleep duration and insomnia symptoms were compared with participants with adequate sleep durations and without insomnia symptoms.

The test-retest reliability of insomnia symptoms and self-reported sleep duration questions was assessed in a subsample of 205 participants randomly recruited. Considering an interval of 7 to 14 days between the interviews, the reliability was evaluated using Kappa statistics and intraclass correlation coefficient (ICC) with their respective 95% confidence intervals (CI). The ICC for sleep duration was good (ICC 0.761; 95% CI 0.685 to 0.819). There was a substantial agreement for insomnia, and Kappa values were 0.759 (95% CI 0.651 to 0.867).

#### Cardiometabolic variables

A 12-h fasting blood sample was drawn by venipuncture in the morning (between 7:00 a.m. and 10:00 a.m.) soon after each subject’s arrival at the clinic to measure serum cholesterol, triglycerides, and glycated hemoglobin [35–37]. Participants with glycated hemoglobin levels ≥6.5% were classified in the high glycated hemoglobin group [35]. Hypertriglyceridemia was defined as triglycerides levels ≥150 mg/dL (milligrams per deciliter). HDL level < 40 mg/dL for men and < 50 mg/dL for women defined the low HDL group [35]. Hypertension was defined as a systolic blood pressure ≥ 140 mmHg or, a diastolic blood pressure ≥ 90 mmHg, or the use of medication to treat hypertension [35]. Weight and height were also collected using standard equipment and techniques. Obesity was defined as body mass index (BMI) ≥ 30 kg/m^2^ [35].

#### Sociodemographic and health variables

The questionnaire also provided information on age, sex, education (fundamental, high school or college), menopausal status and leisure physical activity [PA]; this variable was created considering questions related to the frequency, duration and intensity of PA, categorized as follows: 1) none - no PA or some PA but not meeting the other two categories; 2) moderate - ≥3 days of vigorous-intensity PA for at least 20 min/day or ≥ 5 days of moderate-intensity PA and/or walking, in combination or alone, at least 30 min/day or ≥ 5 days of any combination of walking and moderate-or-vigorous-intensity PA achieving a minimum of 600 MET [Metabolic Equivalents]-minutes/week; and 3) high - vigorous-intensity PA on at least 3 days, accumulating a minimum of 1500 MET-minutes/week or ≥ 7 days of any combination of walking and moderate-or-vigorous intensity PA, obtained using the International Physical Activity Questionnaire, (IPAQ long version) [38]. Depression symptoms ascertained using the Clinical Interview Schedule – Revised (CIS– R) [39].

### Statistical analyses

Descriptive analyses were performed to characterize the participants by sex. Given the heterogeneity in creating the range of reference categories for sleep duration, firstly Generalized Additive Models with Bernoulli distribution were used to evaluate the curve shape for the relationship between self-reported sleep duration and the outcomes and to determine the cut-off for hours of sleep duration. The models were fitted considering the potential confounders. After determining the cut-offs for self-reported sleep duration (≤ 6 h; > 6 h - ≤ 8 h; > 8 h), logistic regression analyses were performed to investigate the magnitude of the associations between self-reported sleep duration, insomnia symptoms and the cardiometabolic risk factors (binary outcomes - high glycated hemoglobin levels, obesity, hypertriglyceridemia, hypertension and low HDL levels). The analyses were adjusted for potential confounders. Model 1 tested a crude association. Then, the analyses were adjusted for age and education (model 2) and included physical activity and depression symptoms (model 3). The fourth model was adjusted for self-reported sleep duration or insomnia symptoms to investigate independent effects. The last model included menopausal status. Joint associations of insomnia symptoms and self-reported sleep duration with all outcomes were examined considering participants with adequate sleep duration (> 6 h - ≤ 8 h) and absence of insomnia symptoms as the reference group.

All analyses were performed using the software R version 2.15.

## Results

Participants in the ELSA-Brasil included 7491 women and 6231 men in this study. For women and men, the mean age was 55.6 years and 55 years. In relation to sleep patterns, 47.6 and 49.1% of women and men, respectively, reported short sleep duration; 4.5 and 3.2%, respectively, reported long sleep duration; and 27.8 and 19.3% of women and men, respectively, reported insomnia symptoms. For both women and men, a higher proportion of individuals with self-reported adequate sleep durations was observed among those with a high level of education, high level of physical activity, and obesity and those without depressive symptoms. The group with insomnia symptoms was more likely to include participants with a low level of education, with no physical activity and with depressive symptoms (Table 1).

**Table 1:** Sleep duration and insomnia symptoms according to sociodemographic and cardiometabolic risk factors by sex. Brazilian Longitudinal Study of Adult Health (ELSA-Brasil, 2012–2014)

	Women (***N*** = 7491)					Men (***N*** = 6231)				
	Self-reported Sleep Duration			Insomnia Symptoms		Self-reported Sleep Duration			Insomnia Symptoms	
	≤ 6 h (*n* = 3564)	> 6 h - ≤ 8 h (*n* = 3592)	> 8 h (*n* = 335)	No (*n* = 5406)	Yes (*n* = 2085)	≤ 6 h (*n* = 3063)	> 6 h - ≤ 8 h (*n* = 2968)	> 8 h (*n* = 200)	No (*n* = 5031	Yes (*n* = 1200)
**Age** (year)*	55.7 (8.8)	55.4 (8.6)	57.8 (9.6)	55.6 (8.7)	56.1 (8.8)	55.5 (8.9)	55.8 (9.5)	56.6 (9.4)	55.6 (9.2)	56.3 (9.2)
**Education*n*(%)**										
Fundamental	332 (9.3)	237 (6.6)	50 (14.9)	358 (6.6)	261 (12.5)	473 (15.4)	385 (13.0)	50 (24.9)	657 (13.1)	251 (20.9)
High school	1235 (34.6)	1041 (29.0)	134 (40.0)	1675 (31.0)	735 (35.3)	967 (31.6)	859 (28.9)	78 (38.9)	1509 (30.0)	395 (32.9)
College	1997 (56.1)	2314 (64.4)	151 (45.1)	3373 (62.4)	1089 (52.2)	1623 (53.0)	1724 (58.1)	72 (36.2)	2865 (56.9)	554 (46.2)
**Physical activity*n*(%)**										
No	2848 (79.9)	2694 (75.0)	278 (83.0)	4116 (76.1)	1704 (81.7)	2199 (71.8)	2008 (67.6)	150 (75.1)	3481 (69.2)	876 (73.0)
Moderate	514 (14.2)	675 (18.8)	44 (13.1)	946 (17.5)	287 (13.8)	541(17.7)	632 (21.3)	28 (13.9)	984 (19.6)	217 (18.1)
High	202 (5.7)	223 (6.2)	13 (3.9)	344 (6.4)	94 (4.5)	323 (10.5)	328 (11.1)	22 (11.0)	566 (11.2)	107 (8.9)
**BMI*n*(%)**										
Non obese	2436 (68.4)	2677 (74.5)	208 (62.1)	3927 (72.6)	1394 (66.9)	2343 (76.5)	2293 (77.3)	146 (73.0)	3863 (76.8)	919 (76.6)
Obese	1128 (31.6)	915 (25.5)	127 (37.9)	1479 (27.6)	691 (33.1)	720 (23.5)	675 (22.7)	54 (27.0)	1168 (23.2)	281 (23.4)
**Low HDL*n*(%)**										
No	2556 (71.7)	2694 (75.0)	233 (69.6)	4017 (74.3)	1466 (70.3)	2388 (77.9)	2324 (78.3)	157 (78.5)	3928 (78.1)	941 (78.4)
Yes	1008 (28.3)	898 (25.0)	102 (30.4)	1389 (25.7)	619 (29.7)	675 (22.1)	644 (21.7)	43 (21.5)	1103 (21.9)	259 (21.6)
***Hypertriglyceridemia*n(%)**										
No	2787 (78.2)	2868 (79.8)	254 (75.8)	4325 (80.0)	1584 (76.0)	1996 (65.2)	1922 (64.8)	105 (52.5)	3278 (65.2)	745 (62.1)
Yes	777 (21.8)	724 (20.2)	81 (24.2)	1081 (20.0)	501 (24.0)	1067 (34.8)	1046 (35.2)	95 (47.5)	1753 (34.8)	455 (37.9)
**High glycated hemoglobin*n*(%)**										
No	3278 (91.9)	3375 (93.9)	294 (87.8)	5066 (93.7)	1881 (90.2)	2765 (90.3)	2676 (90.2)	180 (89.9)	4559 (90.6)	1062 (88.5)
Yes	286 (8.1)	217 (6.1)	41 (12.2)	340 (6.3)	204 (9.8)	298 (9.7)	292 (9.8)	20 (10.1)	472 (9.4)	138 (11.5)
**Hypertension*n*(%)**										
No	2151 (60.3)	2381 (66.3)	169 (50.4)	3513 (65.0)	1188 (57.0)	1682 (54.9)	1652 (55.7)	102 (51.1)	2814 (55.9)	622 (51.8)
Yes	1413 (39.7)	1211 (33.7)	166 (49.6)	1893 (35.0)	897 (43.0)	1381 (45.1)	1316 (44.3)	98 (48.9)	2217 (44.1)	578 (48.2)
**Depressive symptoms*n*(%)**										
No	2782 (78.1)	3101 (86.3)	283 (84.5)	4800 (88.8)	1366 (65.5)	2714 (88.6)	2757 (92.9)	180 (90.0)	4702 (93.5)	949 (79.1)
Yes	782 (21.9)	491(13.7)	52 (15.5)	606 (11.2)	719 (34.5)	211 (11.4)	349 (7.1)	20 (10.0)	329 (6.5)	251 (20.9)

* mean (standard deviation)

**Fig. 1:**
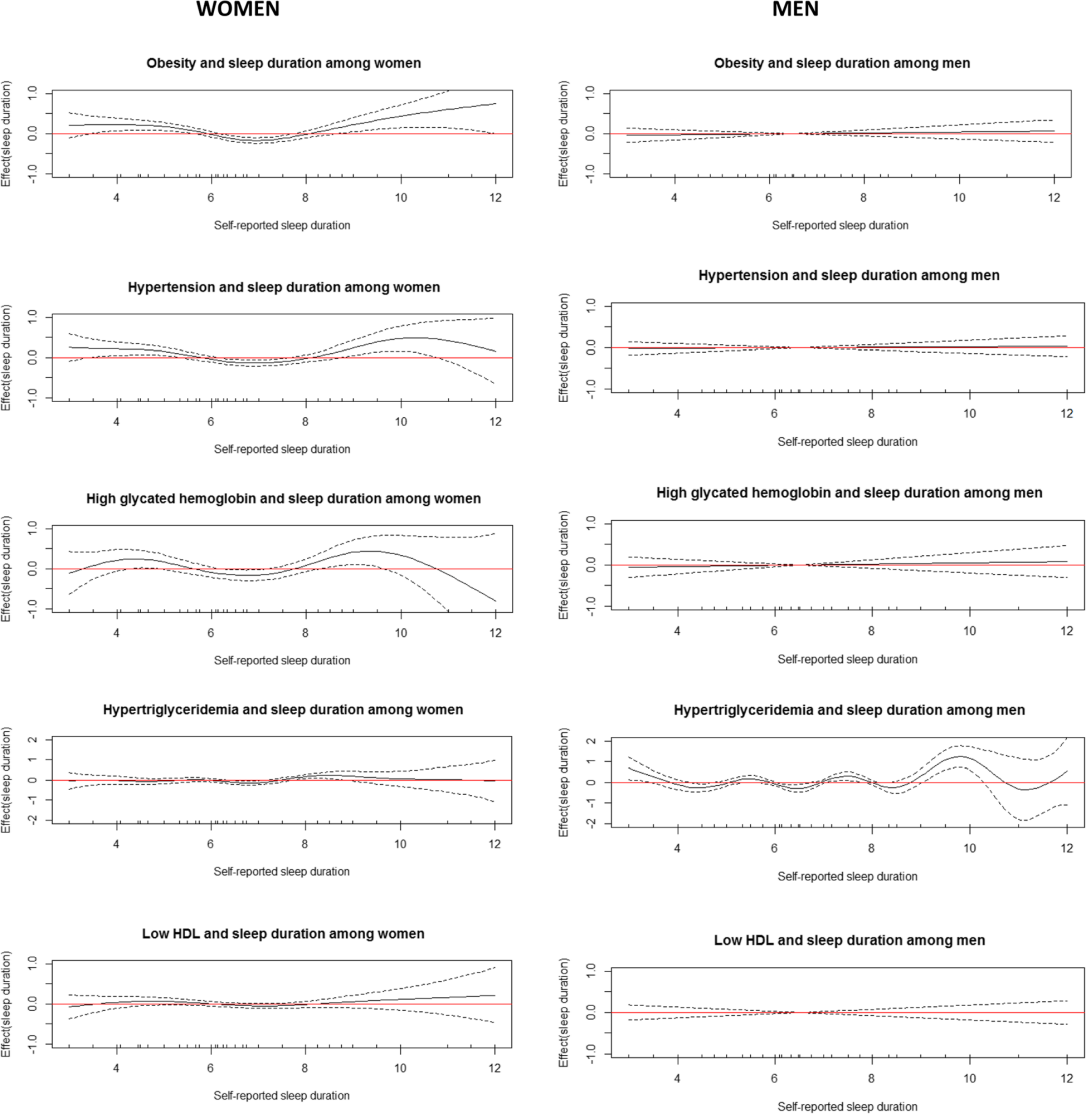
Cardiometabolic risk factors by self-reported sleep duration. ELSA-Brasil (2012–2014). Generalized Additive Models (adjusted for all potential confounders) were used to evaluate the curve shape for the relationship between self-reported sleep duration and the outcomes. The solid black line represents the regression line and the dotted lines represent the 95% confidence interval. The horizontal red line indicates that there is no association

For women, a U-shaped relationship was suggested between self-reported sleep duration and obesity, hypertension and glycated hemoglobin after adjusting for the confounders; adequate self-reported sleep duration (> 6 h - ≤ 8 h) was not associated with cardiometabolic risk factors. In contrast, no significant association between self-reported sleep duration and the outcomes was observed for men, except for hypertriglyceridemia, which seems to be related mainly to long sleep duration (Fig. 1).

For women, in age-adjusted analyses revealed that self-reported sleep duration and insomnia symptoms were significantly associated with obesity, hypertension, low HDL, and high glycated hemoglobin levels compared to the respective reference groups. After adjusting for all potential confounders, the associations remained statistically significant for obesity, hypertension, and high glycated hemoglobin level, and the latter showed the strongest association. Also in women, it should be noted that for obesity, hypertension and high glycated hemoglobin levels, the magnitudes of the associations with the combination of self-reported short sleep duration with insomnia symptoms were higher than those observed for the factors analyzed separately (Table 2). As previously highlighted in Fig. 1, these associations were not statistically significant for men, except for hypertriglyceridemia; both long sleep duration and insomnia symptoms were associated with hypertriglyceridemia after adjusted for the confounders (Table 3).

**Table 2:** Association (odds ratio [95%CI]) between sleep patterns and cardiometabolic risk factors among women. Brazilian Longitudinal Study of Adult Health (ELSA-Brasil, 2012–2014)

	Obesity				
	Model 1	Model 2	Model 3	Model 4	Model 5
**Self-reported sleep duration**					
≤ 6 h	**1.35 (**1.22;1.50)	**1.29** (1.16;1.43)	**1.25** (1.13;1.39)	**1.23** (1.10;1.37)	**1.22** (1.09;1.36)
> 6 - ≤ 8 h	Reference category				
> 8 h	**1.79** (1.41;2.25)	**1.59** (1.25;2.01)	**1.55** (1.22;1.97)	**1.56** (1.23;1.97)	**1.56** (1.23;1.98)
**Insomnia symptoms**					
No	Reference category				
Yes	**1.32** (1.18;1.47	**1.23** (1.10;1.38)	**1.15** (1.02;1.29)	1.09 (0.97;1.23)	1.09 (0.96;1.23)
**Short sleep + insomnia**					
No (> 6 - ≤ 8 h and absence of insomnia)	Reference category				
Yes	**1.58** (1.38;1.81)	**1.43** (1.25;1.65)	**1.32** (1.14;1.53)	**–**	**1.31** (1.13;1.51)
	**Hypertension**				
**Self-reported sleep duration**					
≤ 6 h	**1.29** (1.17;1.42)	**1.29** (1.17;1.43)	**1.19** (1.08;1.33)	**1.15** (1.03;1.28)	**1,15** (1.03;1.28)
> 6 - ≤ 8 h	Reference category				
> 8 h	**1.93** (1.54;2.42)	**1.73** (1.36;2.19)	**1.52** (1.19;1.93)	**1.53** (1.20;1.94)	**1.53** (1.20;1.95)
**Insomnia symptoms**					
No	Reference category				
Yes	**1.40** (1.26;1.55)	**1.39** (1.24;1.54)	**1.22** (1.09;1.37)	**1.19** (1.05;1.33)	**1.18** (1.05;1.33)
**Short sleep + insomnia**					
No (> 6 - ≤ 8 h and absence of insomnia)	Reference category				
Yes	**1.62** (1.43;1.84)	**1.46** (1.28;1.67)	**1.37** (1.19;1.58)	**–**	**1.33** (1.15;1.53)
	**High glycated hemoglobin**				
**Self-reported sleep duration**					
≤ 6 h	**1.36** (1.13;1.63)	**1.35** (1.12;1.62)	**1.20** (1.00;1.45)	1.12 (0.92;1.36)	1.11 (0.91;1.36)
> 6 - ≤ 8 h	Reference category				
> 8 h	**2.17** (1.50;3.06)	**1.90** (1.31;2.70)	**1.62** (1.11;2.32)	**1.64** (1.13;2.34)	**1.65** (1.13;2.23)
**Insomnia symptoms**					
No	Reference category				
Yes	**1.62** (1.35;1.94)	**1.59** (1.31;1.89)	**1.32** (1.09;1.60)	**1.31** (1.06;1.61)	**1.30** (1.06;1.60)
**Short sleep + insomnia**					
No (> 6 - ≤ 8 h and absence of insomnia)	Reference category				
Yes	**1.91** (1.52;2.39)	**1.60** (1.27;2.01)	**1.48** (1.17;1.88)	**–**	**1.43** (1.12;1.82)
	**Hypertriglyceridemia**				
**Self-reported sleep duration**					
≤ 6 h	1.10 (0.99;1.24)	1.10 (0.98;1.23)	1.05 (0.93;1.17)	1.04 (0.89;1.13)	0.99 (0.88;1.12)
> 6 - ≤ 8 h	Reference category				
> 8 h	1.26 (0.96;1.64)	1.20 (0.91;1.55)	1.11 (0.84;1.44)	1.11 (0.85;1.44)	1.12 (0.86;1.46)
**Insomnia symptoms**					
No	Reference category				
Yes	**1.27** (1.12;1.43)	**1.25** (1.11;1.41)	**1.17** (1.03;1.33)	**1.17** (1.03;1.34)	**1.16** (1.01;1.32)
**Short sleep + insomnia**					
No (> 6 - ≤ 8 h and absence of insomnia)	Reference category				
Yes	**1.31** (1.13;1.52)	**1.23** (1.06;1.43)	**1.18** (1.01;1.38)	**–**	**1.12** (0.95;1.31)
	**Low HDL-cholesterol**				
**Self-reported sleep duration**					
≤ 6 h	**1.18** (1.06;1.31)	**1.19** 1.07;1.32)	**1.13** (1.01;1.25)	1.10 (0.99;1.23)	1.10 (0.98;1.23)
> 6 - ≤ 8 h	Reference category				
> 8 h	**1.31** (1.03;1.67)	**1.36** (1.06;1.74)	1.24 (0.97;1.59)	1.25 (0.97;1.59)	1.25 (0.97;1.59)
**Insomnia symptoms**					
No	Reference category				
Yes	**1.22** (1.09;1.36)	**1.23** (1.10;1.38)	**1.13** (1.00;1.27)	1.10 (0.97;1.25)	1.10 (0.96;1.25)
**Short sleep + insomnia**					
No (> 6 - ≤ 8 h and absence of insomnia)	Reference category				
Yes	**1.33** (1.16;1.53)	**1.27** (1.10;1.46)	**1.21** (1.04;1.39)	–	**1.19** (1.03;1.38)

Logistic regression analyses (odds ratio and 95% CI). Model 1 = crude model; Model 2 = Model1 + age + education; Model 3 = Model 2 + physical activity + depression; Model 4 = Model 3 + insomnia or sleep duration; Model 5 = Model 4 + menopausal status

**Table 3:** Association (odds ratio [95%CI]) between sleep patterns and cardiometabolic risk factors among men. Brazilian Longitudinal Study of Adult Health (ELSA-Brasil, 2012–2014)

	Obesity			
	Model 1	Model 2	Model 3	Model 4
**Self-reported sleep duration**				
≤ 6 h	1.04 (0.93;1.18)	1.03 (0.91;1.16)	1.02 (0.90;1.15)	1.02 (0.90;1.16)
> 6 - ≤ 8 h	Reference category			
> 8 h	1.26 (0.90;1.73)	1.22 (0.87;1.62)	1.20 (0.86;1.65)	1.06 (0.86;1.65)
**Insomnia symptoms**				
No	Reference category			
Yes	1.01 (0.87;1.17)	1.00 (0.86;1.16)	0.99 (0.85;1.15)	0.99 (0.84;1.16)
**Short sleep + insomnia**				
No (> 6 - ≤ 8 h and absence of insomnia)	Reference category			
Yes	1.07 (0.89;1.27)	1.04 (0.87;1.27)	1.02 (0.85;1.23)	**–**
	**Hypertension**			
**Self-reported sleep duration**				
≤ 6 h	1.03 (0.93;1.14)	1.04 (0.94;1.16)	1.03 (0.93;1.15)	1.02 (0.91;1.14)
> 6 - ≤ 8 h	Reference category			
> 8 h	1.19 (0.89;1.59)	1.06 (0.78;1.44)	1.06 (0.78;1.43)	1.06 (0.78;1.43)
**Insomnia symptoms**				
No	Reference category			
Yes	**1.18** (1.04;1.34)	1.09 (0.96;1.25)	1.07 (0.94;1.23)	1.07 (0.93;1.23)
**Short sleep + insomnia**				
No (> 6 - ≤ 8 h and absence of insomnia)	Reference category			
Yes	**1.21** (1.04;1.41)	1.13 (0.97;1.33)	1.11 (0.94–1.30)	**–**
	**High glycated hemoglobin**			
**Self-reported sleep duration**				
≤ 6 h	0.99 (0.83;1.17)	0.97 (0.82;1.15)	0.95 (0.79;1.12)	0.93 (0.78;1.11)
> 6 - ≤ 8 h	Reference category			
> 8 h	1.07 (0.65;1.67)	0.88 (0.53;1.39)	0.87 (0.53;1.37)	0.87 (0.53;1.37)
**Insomnia symptoms**				
No	Reference category			
Yes	**1.25** (1.02;1.53)	1.11 (0.90;1.36)	1.04 (0.84;1.28)	1.06 (0.85;1.32)
**Short sleep + insomnia**				
No (> 6 - ≤ 8 h and absence of insomnia)	Reference category			
Yes	1.19 (0.93;1.51)	1.05 (0.82;1.34)	0.98 (0.75;1.25)	**–**
	**Hypertriglyceridemia**			
**Self-reported sleep duration**				
≤ 6 h	0.98 (0.88;1.09)	0.97 (0.87;1.08)	0.96 (0.86;1.07)	0.93 (0.84;1.04)
> 6 - ≤ 8 h	Reference category			
> 8 h	**1.65** (1.24;2.19)	**1.60** (1.20;2.14)	**1.59** (1.19;2.13)	**1.60** (1.20;2.14)
**Insomnia symptoms**				
No	Reference category			
Yes	0.98 (0.84;1.14)	1.13 (0.99;1.29)	1.13 (0.99;1.29)	**1.17** (1.02;1.35)
**Short sleep + insomnia**				
No (> 6 - ≤ 8 h and absence of insomnia)	Reference category			
Yes	1.14 (0.97;1.33)	1.12 (0.96;1.31)	1.11 (0.94;1.30)	**–**
	**Low HDL-cholesterol**			
**Self-reported sleep duration**				
≤ 6 h	1.02 (0.90;1.15)	1.01 (0.89;1.14)	0.99 (0.88;1.12)	1.01 (0.89;1.14)
> 6 - ≤ 8 h	Reference category			
> 8 h	0.98 (0.69;1.38)	0.95 (0.66;1.33)	0.93 (0.65;1.31)	0.93 (0.65;1.31)
**Insomnia symptoms**				
No	Reference category			
Yes	0.98 (0.84;1.14)	0.96 (0.83;1.12)	0.93 (0.80;1.09)	0.93 (0.79;1.09)
**Short sleep + insomnia**				
No (> 6 - ≤ 8 h and absence of insomnia)	Reference category			
Yes	1.01 (0.84;1.21)	0.98 (0.82;1.18)	0.94 (0.78;1.13)	–

Logistic regression analyses (odds ratio and 95% CI). Model 1 = crude model; Model 2 = Model 1 + age + education; Model 3 = Model 2 + physical activity + depression; Model 4 = Model 3 + insomnia or sleep duration

## Discussion

Results suggest sex-specific patterns, since obesity, hypertension and high glycated hemoglobin were associated with self-reported sleep duration and insomnia symptoms (either separately or linked to short sleep duration) in women, but not in men. In addition, cardiometabolic risk factors were associated with insomnia symptoms plus short sleep duration only in women. In relation to hypertriglyceridemia, statistically significant associations with insomnia symptoms were observed among both women and men.

Observational studies have shown that the prevalence of insomnia symptoms varies from 10 to 30% [3, 21, 25], and similar to our findings, insomnia symptoms are more frequent among women than men [17, 25, 30]. This difference is more evident in self-reported data than objective measures [26]. Regarding sleep duration, our findings indicated that almost half of the sample reported short sleep duration. This prevalence was higher than those observed in other countries [11, 21, 31]. However, for long sleep duration, our findings are in agreement with previous studies [31, 22].

### Sex-specific patterns considering short sleep duration

Some studies reported significant associations between sleep problems and cardiometabolic outcomes for women but not men. Similar to our findings, in the Whitehall cohort, short sleep duration was associated with a higher risk of hypertension only in women [28]. Also, Grandner et al. [40] evaluated the association between hypertension and sleep duration in a large survey population and observed that short sleep was more closely related to hypertension in women than men. A meta-analysis of the association between sleep duration and hypertension revealed that the results from cross-sectional studies suggested statistically significant associations between short sleep duration and hypertension in women but not in men [8].

In relation to body weight, Westerlund et al. [30] observed higher BMI values in women and men with short sleep duration compared with adequate sleep duration (6–8 h). On the other hand, similar to our findings, short sleep duration was associated with a greater BMI among women but not among men [31]. A recent meta-analysis [6] showed an association between short sleep duration and the risk for obesity (lowest risk at 7–8 h sleep/day), stressing that the associations seem to be more evident in women than men. However, conclusions were not yet possible given the few studies addressing possible gender differences [6]. The same limitation was pointed out by Shan et al. [5], who found an association between short sleep duration and an increased risk for type 2 diabetes in a meta-analysis of prospective studies with adult populations. In contrast, our findings on the association between short sleep duration and high glycated hemoglobin were not statistically significant in women or men, although a greater tendency for a positive association was observed among women. Regarding plasma lipids, similar to our results, Kim et al. [41] did not identify associations between short sleep duration and dyslipidemia in women or men. However, meta-analyses highlight the lack of sufficient evidence to support any conclusion on this issue [10, 29].

### Sex-specific patterns considering long sleep duration

Westerlund et al. [30] also observed higher BMI values in women with long sleep duration. In contrast, long sleep duration was associated with lower BMI values in men. Kim et al. [41] observed significant associations of long sleep duration with central obesity, low HDL, and high fasting glucose levels among women but not among men. According to our data, long sleep duration was associated with elevated triglyceride levels only among men [41], which deserves further investigations. As mentioned above, recent meta-analyses have addressed the association between sleep duration and cardiometabolic risk factors, but sex-differences approaches are scarce. A reverse J-shaped relationship was described in a recent meta-analysis, with a borderline association between long sleep duration and the risk of obesity [6]. A meta-analysis of longitudinal studies that examined the association between sleep duration and diabetes showed that long sleep duration were associated with a significantly increased risk of diabetes compared with 7–8 h of sleep/day [5].

Concerning the U-shaped relationship between sleep duration and hypertension, a meta-analysis found that the results from prospective studies showed that neither short nor long sleep duration was statistically associated with the risk of incident hypertension [8]. However, similar to our findings for women, analyses from cross-sectional studies showed that both short and long sleep durations were associated with prevalent hypertension in adults [8]. Another meta-analysis of cohort studies showed significant associations between short sleep duration and higher risk of hypertension [3]. However, the authors found no statistical evidence of any effect of long sleep duration on the risk of hypertension [3]. A meta-analysis of prospective studies identified that short sleep duration was significantly associated with increased risk of obesity, whereas long sleep duration had no effects on obesity [16]. A systematic review with meta-analyses examining the relationship between short sleep duration and several health outcomes identified significant results for diabetes, hypertension, cardiovascular disease and obesity [11]. The results from this same research group showed that long sleep duration was significantly associated with diabetes and obesity, but not with increased risk of hypertension [12]. In summary, the literature is not entirely consistent with respect to the U-shaped associations between sleep duration and metabolic outcomes. Some results from meta-analyses including longitudinal studies have showed statistically significant associations of both short and long sleep duration with diabetes and obesity [5, 12], while other studies that evaluated hypertension and obesity did not identify any associations [3, 16]. Part of these disagreements could be attributed to methodological issues, such as the instrument used to assess sleep duration and the outcomes (subjective or objective measure), the cut-offs for sleep duration, and the duration of follow-up [3].

### Sex-specific patterns considering insomnia symptoms

Sex-specific patterns concerning the association between insomnia symptoms and cardiometabolic factors are scarce. Cross-sectional analyses [30] showed higher BMI values in women with insomnia symptoms, and a similar pattern but less marked among men. In contrast to our findings, insomnia was associated with a hypertriglyceridemia in women, but significant results were not found in men [29]. Also, difficulty maintaining sleep was associated with an increased overall risk for cardiovascular events in women, but not men [42].

Regarding data from the general population without discussion of sex-specificities, insomnia symptoms were associated with hypertension medication [43], dyslipidemia medication [43], and type 2 diabetes [7]. A meta-analysis of cohort studies showed that insomnia symptoms were significantly associated with a higher risk of hypertension [3]. However, among individuals with adequate sleep duration, insomnia symptoms were not associated with obesity [22].

### Joint associations of insomnia and short sleep duration

A recent study found a significant association between insomnia symptoms and obesity among participants with short sleep duration, but not among those who reported adequate sleep duration [22]. In the same way, short sleep duration with insomnia symptoms was associated with hypertension [23] and diabetes [24]. In a cross-sectional study, associations between sleep duration and BMI were not modified by insomnia symptoms [30].

Our findings on the associations between self-reported sleep duration and each outcome independent of insomnia symptoms (and vice versa) also deserve attention. It has been discussed that insomnia and short sleep duration are distinct aspects of sleep that exhibit intersections. We did not find distinct results after adjusting for insomnia or sleep duration, i.e., the magnitude of the association was similar regardless of the adjustment for the other sleep variable. However, the combination of insomnia symptoms and self-reported short sleep duration appears to intensify the adverse effects of sleep problems separately from the independent associations [20, 21]. Therefore, in line with previous studies [23, 24], we found that the magnitudes of the associations between self-reported short sleep duration plus insomnia symptoms and the outcomes were slightly increased. These findings stress the relevance of considering sleep problems as an additional risk of disease.

### Possible explanations and mechanisms

In line with our findings, Prather et al. [27] showed that associations between sleep quality and biomarkers were stronger in women than in men. Poorer sleep quality was associated with 5-years increases in Interleukin 6, C-reactive protein, and fibrinogen in women but not in men. Therefore, causal pathways may be different for men and women. Therefore, causal pathways may be different for men and women. A laboratory study examining the effects of sleep deprivation on neural cardiovascular control in men and women found that sleep deprivation decreased muscle sympathetic nerve activity only in men, which in turn exerted a protective function on blood pressure [44]. Concerning differences in levels of reproductive hormones, it is possible that testosterone, which is higher in men, may attenuate the effects of poor sleep on inflammation. Supporting this hypothesis, lower circulating levels of estradiol contribute to the increased levels of inflammatory activity associated with poor sleep in women around the menopausal period (~ 55 years) [27]. The association between sleep problems and greater psychological distress in women but not in men [26] could also explain these differences. Greater psychological distress in women is generally attributed to a more stressful role for women in Western societies.

Although sex-specific association between sleep and cardiometabolic problems represents an important research area [5, 6, 32], the mechanisms underlying the different patterns are not completely elucidated because few studies have investigated sex differences related to this issue [41]. Therefore, further studies on sex differences and more mechanism studies are needed to clarify this issue.

In a general way, the biological evidence supporting a putative mechanism between sleep deprivation and cardiometabolic outcomes is derived from disturbances in autonomic function, and inflammatory and hormonal profiles [33]. For instance, sleep reduction affects energy balance, increases energy intake and reduces energy expenditure. Nocturnal awakening is also associated with altered leptin levels that lead to leptin resistance and result in glucose impairment. Elevated appetite could lead to weight gain and in turn, increases insulin resistance [5, 32]. Possible pathways for the association between long sleep duration and cardiometabolic disease are not clear. Some authors discuss the possibility that long sleepers are previously sick individuals, and for that reason, these associations should be observed in this group. Long sleep is also associated with others sleep disorders, such as obstructive sleep apnea, and poor-quality sleep (increased sleep fragmentation and more frequent awakenings), which lead to changes in inflammatory markers that have been shown to be associated with metabolic dysregulation [17, 33, 45]. Disagreeing with the hypothesis that morbidity leads to long sleep duration, some studies stress the significant associations of long sleep duration with mortality, diabetes and other diseases, after controlling for other comorbidities [17, 45, 46]. It should be noted that in the study of the joint associations of insomnia and sleep duration with diabetes, the adjustment for body mass index did not attenuate associations of long sleep duration without insomnia and diabetes [17]. In this way, a sensitivity analysis by exclusion of the participants diagnosed with myocardial infarction, stroke, and cancer during the first 2 years of follow-up did not change the association between long sleep duration and diabetes [45]. Clearly, there is a demand for further studies on long sleepers.

### Strengths and weakness

The strengths of this study are the large multicenter sample of middle-aged Brazilians, the use of standard equipment and techniques and the rigorous quality control of interviews and all measurements. All outcomes included were evaluated by objective measures. Also, we investigated the relationship between sleep duration and the cardiometabolic risk factors, examining firstly if the shape of these possible associations was linear or nonlinear in order to determine the cut off points for sleep duration based on the patterns of our sample.

The limitations of this study include the fact that all sleep variables were self-reported. Also, the insomnia symptoms did not include information related to daytime dysfunction. Although measuring objective sleep duration is more accurate, this procedure is generally not feasible in large epidemiological samples [5, 10]. The current study demonstrated substantial reliability for insomnia symptoms and self-reported sleep duration questions. Also, objective sleep-duration measures seemed to be more strongly associated with hypertension than self-reported sleep duration, with no statistically significant differences [3]. Second, we did not have enough information to distinguish participants who were naturally short sleepers (short sleep duration but feel rested) from the others, and there may be differences between those groups. However, the investigation of individuals who reported short sleep duration plus insomnia symptoms showed results in the same direction of those observed for short sleepers. Therefore, we supposed that most individuals who reported short sleep duration do not have high sleep quality. Third, although the cross-sectional study design did not allow us to infer causality, the literature on sleep and metabolic disorders suggests that sleep problems most likely enhance the probability of developing cardiometabolic outcomes [24]. Fourth, our plasma measures were obtained based on standard procedures between 7:00 a.m. and 10:00 a.m., but the exact moment of assessment was not recorded. As we expect a diurnal variation for some biological markers, the timing of the blood test could be a potential confounder that was not considered. Fifth, we recognize the relevance of age in analysis of cardiometabolic factors. Nevertheless, our sample size (only ~ 4% reported long sleep duration) did not allow us to deeply study age effects – for instances, only 41 individuals with high glycated hemoglobin levels reported long sleep duration). Finally, our study sample comprises a particular population (Brazilian civil servants) and the generalization of our findings should be done with caution.

## Conclusions

Our findings contribute to the discussion on the importance of adequate sleep quality and duration and may have important clinical and public health implications, mainly in relation to primary prevention. It is essential to identify modifiable life factors associated with a lower risk for developing non-communicable diseases, as proposed in the present study. Assessing the associations between sleep problems and cardiometabolic outcomes according to gender contributes to filling the gap in the literature on possible gender differences, highlighted in recent systematic reviews with meta-analyses. The investigation of the lipid profile deserves further attention, since studies on this issue are still rare and inconclusive. The study contributes to the recognition that sleeping > 6 h to ≤8 h per night can be treated as presenting the lowest risk for cardiometabolic risk factors. Lastly, the associations of self-reported short sleep duration plus insomnia symptoms with higher likelihoods of obesity, hypertension and high glycated hemoglobin levels reinforces the importance of considering quantitative and qualitative aspects of sleep for the prevention and management of the outcomes.

## Data Availability

The datasets used and analysed during the current study are available upon request to the Study’s Steering Committee, through an appointed representative, Dr. Rosane Harter Griep (rohgriep@gmail.com). The ELSA-Brasil study, while open to any researcher, has a policy of requiring that all proposals of investigations pass through the study ́s publications committee.

## Abbreviations

BMI: Body mass index
CI: Confidence intervals
ELSA-Brasil: Brazilian Longitudinal Study of Adult Health
FIOCRUZ: Oswaldo Cruz Foundation
HDL-cholesterol: High-density lipoprotein cholesterol
ICC: Intraclass correlation coefficient
MET: Metabolic Equivalents
mg/dL: Milligrams per deciliter
PA: Physical activity
UFBA: Federal University of Bahia
UFES: Federal University of Espirito Santo
UFMG: Federal University of Minas Gerais
UFRGS: Federal University of Rio Grande do Sul
USP: Sao Paulo University
